# Reviewing advances in nanophotonic biosensors

**DOI:** 10.3389/fchem.2024.1449161

**Published:** 2024-09-10

**Authors:** Zunaira Javaid, Muhammad Aamir Iqbal, Saher Javeed, Siti Sarah Maidin, Kareem Morsy, Ali A. Shati, Jeong Ryeol Choi

**Affiliations:** ^1^ Department of Biochemistry, Kinnaird College for Women University, Lahore, Pakistan; ^2^ School of Materials Science and Engineering, Zhejiang University, Hangzhou, China; ^3^ Department of Physics, Government College University Lahore, Lahore, Pakistan; ^4^ Faculty of Data Science and Information Technology, INTI International University, Nilai, Malaysia; ^5^ Biology Department, College of Science, King Khalid University, Abha, Saudi Arabia; ^6^ School of Electronic Engineering, Kyonggi University, Suwon, Gyeonggi-do, Republic of Korea

**Keywords:** nanophotonic biosensors, biomolecules, challenges, surface plasmon resonance, phase-driven sensors

## Abstract

Biosensing, a promising branch of exploiting nanophotonic devices, enables meticulous detection of subwavelength light, which helps to analyze and manipulate light-matter interaction. The improved sensitivity of recent high-quality nanophotonic biosensors has enabled enhanced bioanalytical precision in detection. Considering the potential of nanophotonics in biosensing, this article summarizes recent advances in fabricating nanophotonic and optical biosensors, focusing on their sensing function and capacity. We typically classify these types of biosensors into five categories: phase-driven, resonant dielectric nanostructures, plasmonic nanostructures, surface-enhanced spectroscopies, and evanescent-field, and review the importance of enhancing sensor performance and efficacy by addressing some major concerns in nanophotonic biosensing, such as overcoming the difficulties in controlling biological specimens and lowering their costs for ease of access. We also address the possibility of updating these technologies for immediate implementation and their impact on enhancing safety and health.

## 1 Introduction

Biosensors have been shown to have a significant impact on a variety of disciplines, such as food safety, medicine, pharmaceuticals, security, environmental monitoring, and forensics (Chaudhary et al., 2023). In particular, the role of biosensing in diagnostic technology is noteworthy because of the importance of precision in diagnostics, which accounts for over seventy percent of medical checkups. Hospitals and centralized medical research labs frequently collect diagnostic information based on analytical methods that utilize biosensing techniques, such as light microscopy, labeled immunoassays, and cell-culture tests (Ngo et al., 2017). The recognition of target analytes in the case of labeled immunoassays among them can be facilitated by enzyme-based tags (Malik et al., 2022a). The applicability of labeled immunoassays is limited and selective, but usually, they are carried out via multi-level detection techniques, which is costly and time-consuming. Exceptionally, a few labeled immunoassays, particularly the lateral-flow assay (LFA) (Ince and Sezgintürk, 2022), are suitable for simple non-clinical applications, while exemplary fields available for such applications are home-based pregnancy tests and COVID-19 testing (Mak et al., 2016; Kevadiya et al., 2021). LFAs can offer measurement results in a few minutes without the need for specific equipment. However, their capacity for sensing is usually limited to a small number of analytes, rendering them semi-quantitative (Kumari et al., 2022; González-Guerrero et al., 2016; Crocetto et al., 2022; Boumaza et al., 2019). Highly epidemic and infectious diseases, which are contagious, pose a significant threat to humanity. Regarding this, early diagnosis may boost the possibility of recovery for infected individuals. In many viral pandemics, like the recent COVID-19 (Chen et al., 2022a), it is critical to develop accessible, accurate, ultrasensitive, and realistically powerful diagnostic techniques for examining the majority of populations. This allows one to examine the way viral transmission occurs and provides critical help in the fight against viral infections. Thus, nanophotonic biosensors can potentially be used to detect viruses, as they can offer more sensitivity, user-friendliness, improved throughput, and compactness, regardless of their performance, when sample numbers in centralized laboratories are considerable and analytical methods for disease treatment are appropriate (Ghio et al., 2023; Zeng et al., 2022).

Early detection is crucial for improving therapy success in inflammatory disorders such as cancer, autoimmune diseases, and rheumatoid arthritis. For example, a liquid biopsy has been used to identify cancer biomarkers (Soda et al., 2019; Byrnes and Weigl, 2018; Toyama and Weissman, 2011). Biofluids have been examined to identify common biomarkers, including prions, cytokines, hormones, antibodies, DNA/RNA, and proteins (Visser et al., 2018; Heikenfeld et al., 2019). This strategy is rapidly being investigated as a non-invasive alternative to surgical biopsies to speed up the detection of certain end-stage illnesses. Most biomarkers may have relatively low concentration levels, meanwhile, it is possible to detect structural changes in biomarker molecules associated with neurodegenerative disorders. The detection of such structural changes is typically a function of the sensitivity and specificity of the detection method. Moreover, ongoing therapeutic biomarker monitoring can assist clinicians in tailoring a patient’s course of treatment (Mak et al., 2016). In addition, bioreceptors can indeed be biomolecules themselves and be used to evaluate biomolecules. They can bind to the surface of the target analyte, which might be an antibody, enzyme, protein, nucleic acid (DNA or RNA), or a synthetic molecule. The whole coordination mechanism between the target analyte and bioreceptor must also be statistically evaluated to develop a biosensor instrument capable of detecting any change in the biological process. There are several ways to identify specific biosensors based on the accessibility of individual bioreceptors, transducers, and combinations of the two. The use of these biosensors allows for the easy recognition of the target analytes, such as pathogens, allergens, or specific molecules associated with infections, allergies, or biomarkers. These signs can help to identify an early diagnosis, which may be useful in monitoring the patient’s health while preserving the patient’s medical history. These tools are quite useful in monitoring the health of any individual, particularly those who are drug addicts or alcoholics. Aside from that, they are useful in supplying information to medical professionals to measure the drug’s amount. Given this, the patient’s molecular profile, which the biosensors may provide becomes crucial in laying the groundwork for precisely the right kind of treatment (Mage et al., 2017; Ho et al., 2020; Ginsburg and Phillips, 2018; Ahmed et al., 2014). Incorporating self-operating and adequate biosensors into public capacity has the potential to simplify people’s safety procedures by preventing health dangers that endanger a healthy existence (Perelló-Roig et al., 2022). The results at the population level can be analyzed utilizing datasets generated, for example, by particular devices connected to the Internet of Things (IoT). High-tech biosensors can be applied to develop an information meshwork based on artificial intelligence algorithms. That is the detection of the analytes, together with analyzing the data associated with the analytes, may help to establish a large amount of data in bioscience (Luan et al., 2018; Wang et al., 2020; Altug et al., 2022; Novotny and Van Hulst, 2011; Iqbal et al., 2023a; Luk’Yanchuk et al., 2010; Bannur et al., 2022; Iqbal et al., 2021; Kuznetsov et al., 2016). Taking this into focus, this review discusses recent advancements that might support this goal in the areas of plasmonic, nanophotonic, and nanostructure-based biosensing devices (Shrivastava et al., 2020; Dey et al., 2019). In particular, we concentrated on improved infrared Raman spectroscopy detection, label-free optical sensing technologies, and the detection of refractive index (RI) changes in biomolecular systems. Furthermore, limitations and emerging innovations have also been covered.

## 2 Nanophotonic biosensors

**FIGURE 1:**
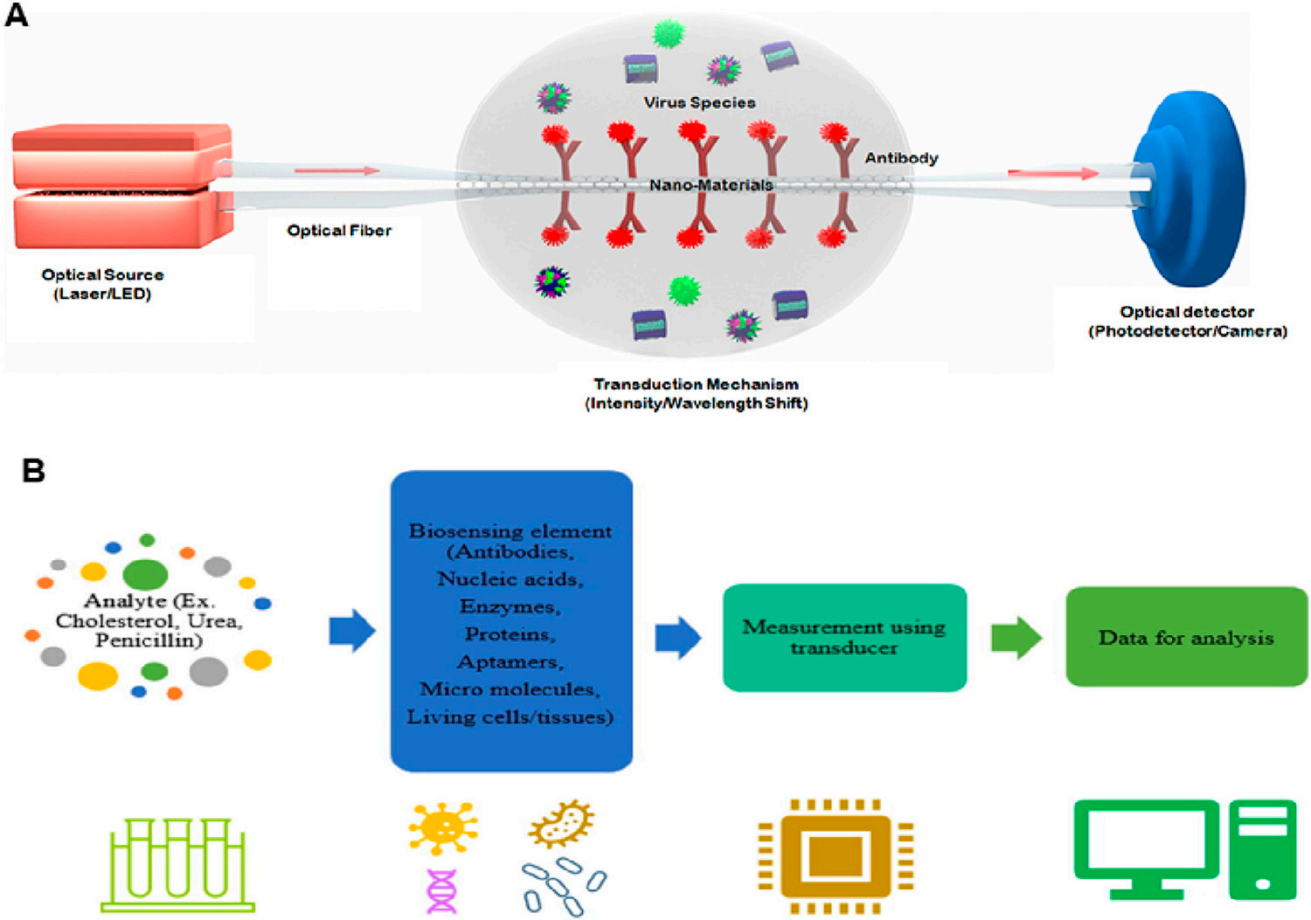
**(A)** Diagrammatic working representation of a standard nanophotonic biosensor detecting viruses (Bannur et al., 2022). **(B)** Important parts of a biosensor, while the analytes may be an antibody, protein, or enzyme (Singh et al., 2023).

The potential applications of nanophotonic devices in biosensing are promising due to their capability to regulate light at subwavelength volumes and improve the interaction of light with matter. To overcome the drawbacks of the existing bioanalytical techniques in terms of sensitivity, user-friendliness, throughput, and compactness, many kinds of nanophotonic biosensors have been developed (Altug et al., 2022). Through the formation of strong electromagnetic field regions, nanophotonic structures can restrict light close to their surface, down to less than 10 nm. They can also act as optical nanoantennas, raising the intensity of localized fields further by 3 to 4 orders of magnitude compared to incoming light (Novotny and Van Hulst, 2011). Such field intensifications increase sensitivity to analyte molecules concurrently but result in decreased susceptibility to external interference. The function of nanophotonic biosensors can be determined by the resonance properties of the nanostructures, characterized by the merging of the evanescent field with the analyte molecules spatially. The transduction platform, optical source, and optical receiver are typical components of a nanophotonic biosensor, as schematically shown in Figure 1A. The transduction platform’s receptors are specifically tailored to meet their optimal connection with the target analytes, such as viruses (Bannur et al., 2022). In the transduction process associated with biosensing, three essential outcomes can happen through the interaction of the target analyte and the light produced by the optical source. (1) a change in the incident light’s refractive index or phase; (2) an adjustment to the light’s intensity or amplitude due to absorption of the incident energy by the biomolecules; or (3) the absorption of energy by biomolecules through the process of Raman scattering or fluorescence, which results in an emission of new light with a different wavelength. Optical phase shifts in substrate platforms can be accomplished through a meticulous design of their optical components. In more detail, phase shifts of light are induced by changing its intensity using interferometers, whereas wavelength shifts are possible using photonic devices such as microresonators and fiber Bragg gratings. The development of viable optical biosensors can be achieved by engineering substrate platforms and devices in a way that they precisely detect changes in the optical characteristics of light, most often its wavelength, phase, or intensity.

**FIGURE 2:**
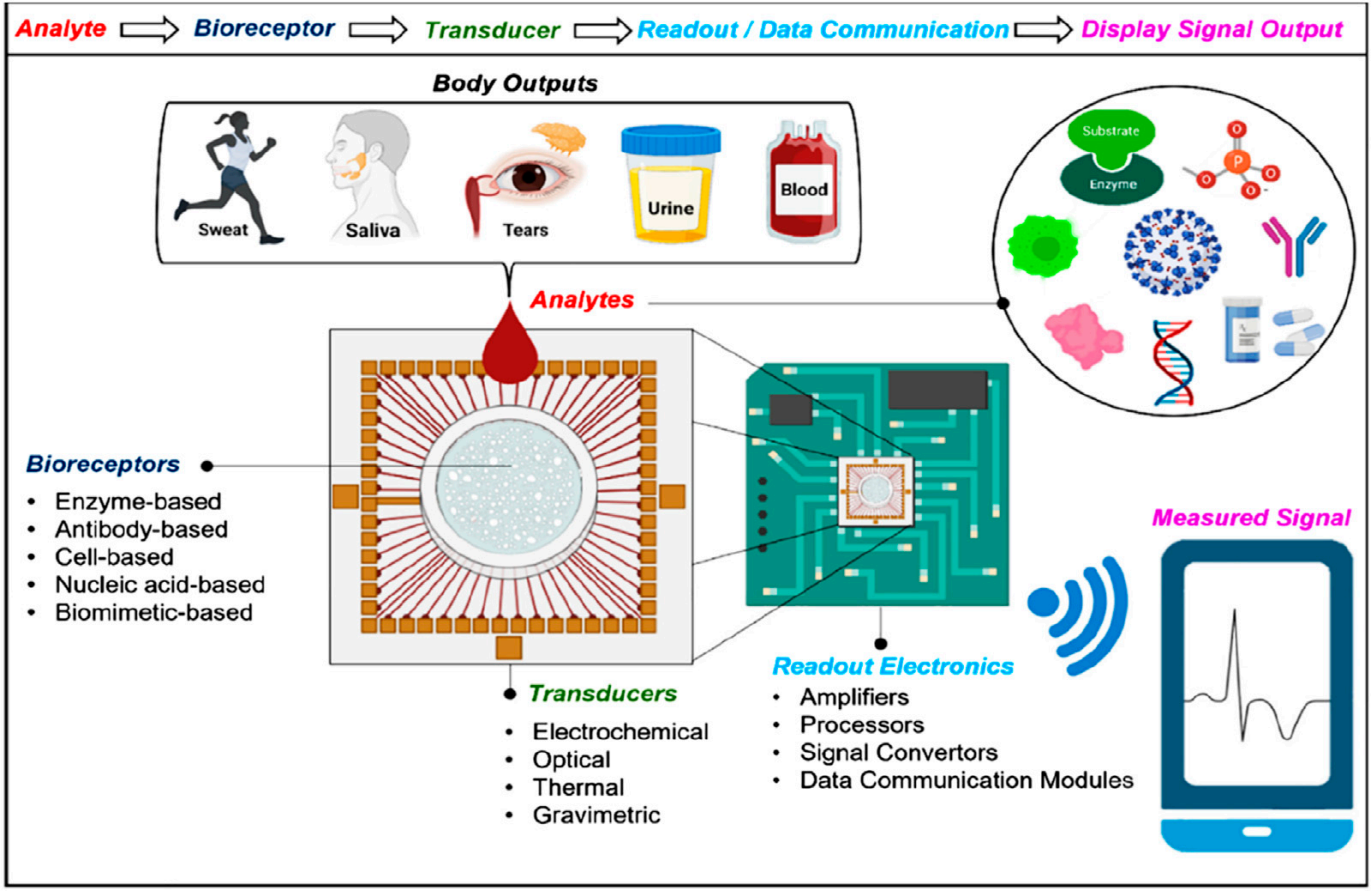
Diagrammatic view of biosensing mechanism. Bio-receptors bind to the surface of the target analyte. Any change in the signal is detected by the transducers, and then the transducers translate it to a quantifiable outcome due to the resemblance between the target analyte and the receptor. The signal can be converted into any other form with the help of light-based, thermal, or electrochemical techniques. The output signal is processed further or transported to the target tool with the help of chip-based or traditional electronics. Recent biosensors have built-in data bank models of communication that help transmit digital data to smart devices. To examine the health of individuals, this enables the derived signals to be portrayed on any device (Polat et al., 2022).

Typical biosensors can examine analytes extracted from various biological liquid samples, including blood, saliva, sweat, and urine as the biological components (Singh et al., 2023; Polat et al., 2022; Chen et al., 2022b). Biosensors have a highly reactive layer as an analytical tool, which distinguishes between a certain transducer and the analyte, producing a signal proportional to the analyte’s concentration. The reactive layer in biosensors interacts with the analyte during the sensing process and produces a message through light emission, proton exchange, heat discharge, assimilation or reflection, ions or gases emission, or any other means. The main function of the transducer is the conversion of a signal into a computative message that can be detected afterward. A good biosensor needs to be designed to handle the physical and chemical demands of long-term operation, including having enough internal capacity for reagents or samples to sustain its function over time. It is required for the sensors to be very selective and to be able to distribute the data with efficient duplicability, accuracy, and reactivity over a wide array of concentrations. The limit of detection (LOD) should be low, and the shelf life should be long. Biomolecule immobilization over the sensing surface of the transducer during the evolution of sensing is the main factor in the biosensing process. The biomolecules or compounds targeted to be detected must be strongly connected to the sensing part of the transducer via the techniques of such an immobilization. A schematic to typically analyze the biosensing mechanism of biomolecules is portrayed in Figure 2 (Polat et al., 2022), while the important components of a biosensor are shown in Figure 1B (Singh et al., 2023). Further, this section is mainly focused on nanophotonic biosensors, such as plasmonic nanostructures, evanescent-field-based biosensors, surface-enhanced spectroscopy biosensors, phase-driven biosensors, and resonant dielectric nanostructures.

### 2.1 Evanescent-field-based biosensors

**FIGURE 3:**
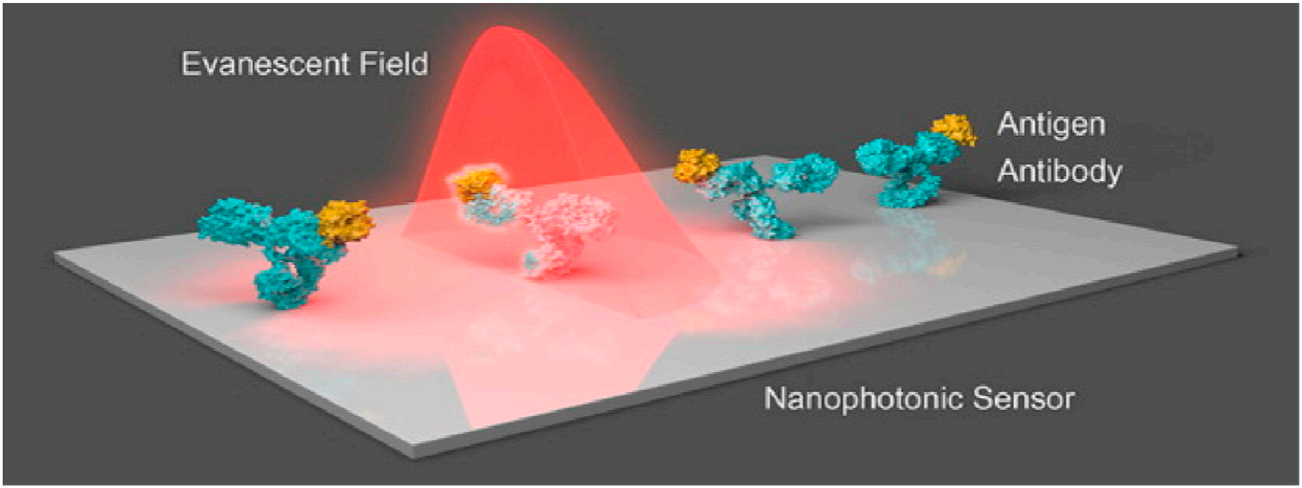
Nanostructures that respond to the incident light field are commonly seen in nanophotonic biosensors. The augmented evanescent field is used to detect changes in refractive index at the interface caused by biorecognition events such as interactions between antibodies and antigens (Soler et al., 2020).

For biomolecules, receptors are being used by optics-based biosensors to propagate with a particular analyte, having an optical component containing a reading system that transforms the communication information into a measurable output. Alternatively, results can be acquired from the optic characteristics of the analyte’s spectroscopic biosensors. Evanescent fields based on photonic biosensors provide a feasible approach for separating and isolating the light-probing analytes present within the liquid samples of optoelectronic components (Chen et al., 2008; Zalyubovskiy et al., 2012; Martinsson et al., 2014). The main characteristics of the evanescent field include the exponential reduction in one direction remaining in parallel to the biosensor surface, with a total length of hundreds of nanometres. The most critical component of the evanescent field is surface concealment, which increases the interactivity of light with matter and, during measurement, brings forth precise spatial grip. The notable prototype of an evanescent-field affinity biosensor is the surface plasmon resonance (SPR) (Mayer and Hafner, 2011; Sönnichsen et al., 2000; Khurgin, 2015; Doiron et al., 2019; Naik et al., 2013), which can take advantage of surface plasmons to distribute over the boundary within the noble metal layer and dielectric layer. The RI of the dielectric medium over the surface of the metal determines the condition, such as momentum-matching, due to which the extrinsic source of light can generate the wave of charge density known as plasmons. With the help of variation calculations in the state of resonance, chemical interactions can be rapidly visualized in real-time, such as in terms of wavelength and intensity. Measurement based on evanescent-field biosensors is a simple, rapid, and non-invasive biochemical process for analyzing target analytes because they can detect biomolecular interactions at the sensor surface in real-time without the necessity of labels or dyes (see Figure 3).

**FIGURE 4:**
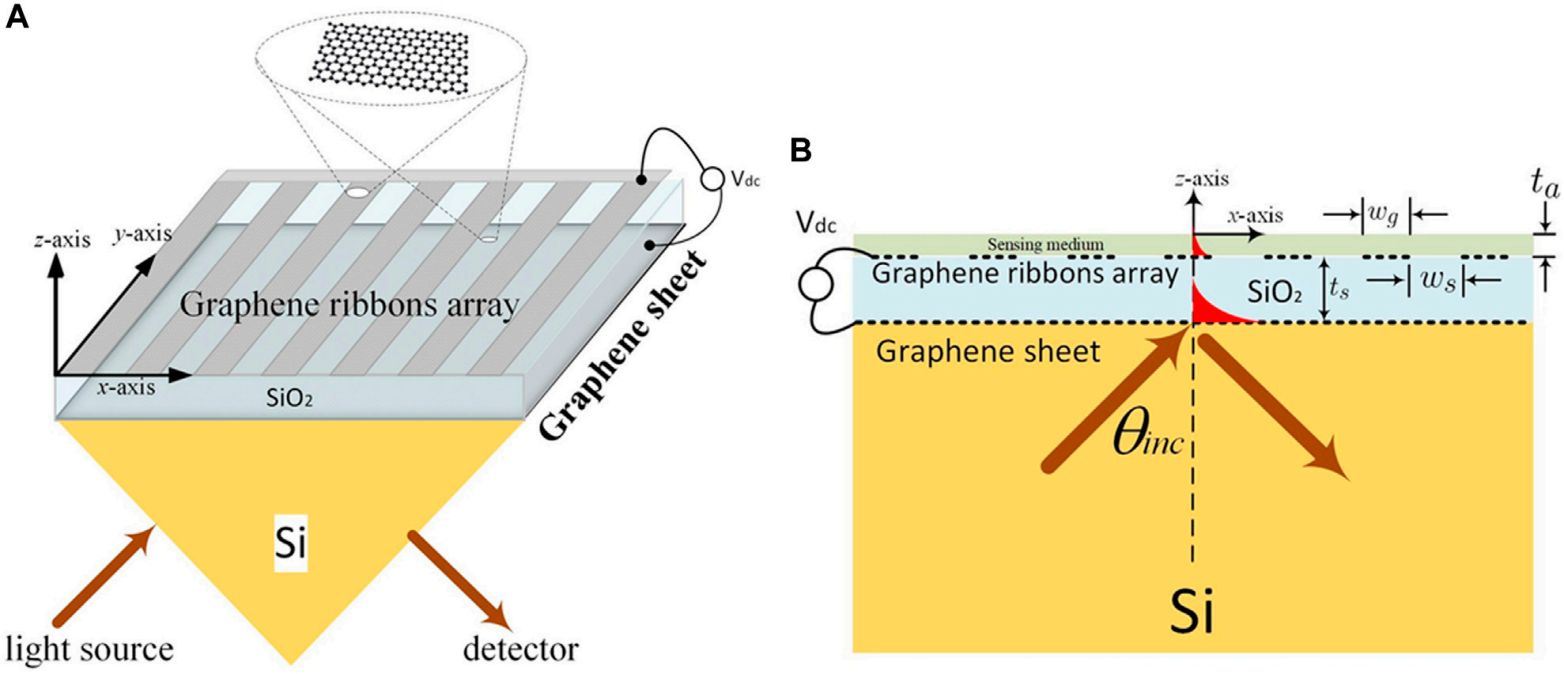
**(A)** Schematic of the evanescent wave-based biosensor, and **(B)** Full-wave electromagnetic analysis with the help of a 2D model (Hwang, 2021).

Resonators and dielectric waveguides are also used in evanescent-field biosensors (Hayashi et al., 1988; Romano et al., 2018a). SPR biosensors, along with the dielectric waveguides, have very sustained decay lengths as compared to other sensors utilizing different mechanisms, while they are available for sensing almost the length of 200–400 nm. Consequently, most of the traditional techniques of the evanescent fields were restricted in exposing the probing light and analyte interactions (Homola and Piliarik, 2006; Hwang, 2021). To maximize a biosensor’s efficiency, Hwang (Hwang, 2021) theoretically investigated an evanescent-wave-based biosensor proposed on graphene-triggered SPRs (see Figure 4), with the incidence of TM-polarization light on the wedge surface (see Figure 4A). He managed the system so that the magnetic and electric fields did not vary along the *y*-axis, leading him to believe that the structure’s size was far larger than the operational wavelength. Then he carried out the scattering analysis using a condensed 2D electromagnetic model (see Figure 4B). By setting the incidence angle (θ_inc_) larger than the critical angle (θ_c_), he projected an electromagnetic plane wave from silica to Si-substrate. Resultantly, the evanescent wave was stimulated, and the entire internal reflection occurred in the silica layer. Additionally, these exponentially decreasing evanescent waves along the *z*-axis stimulated the SPR modes on the graphene ribbons. There is no transmit power measurement since all higher-order space harmonics were below cutoff due to the graphene-ribbon-arrays period (d_x_) being much shorter than the operating wavelength under consideration. In this setup, a periodic array was employed to broaden the detection range. He investigated how the graphene chemical potential influenced the SPR curve’s change and discovered that the sensitivities for analyte RI values of 1.34, 1.33, 1.35, and 1.36 are 40676.5, 36401.1, 40918.2, and 41160 mV/RIU, respectively (Hwang, 2021).

**FIGURE 5:**
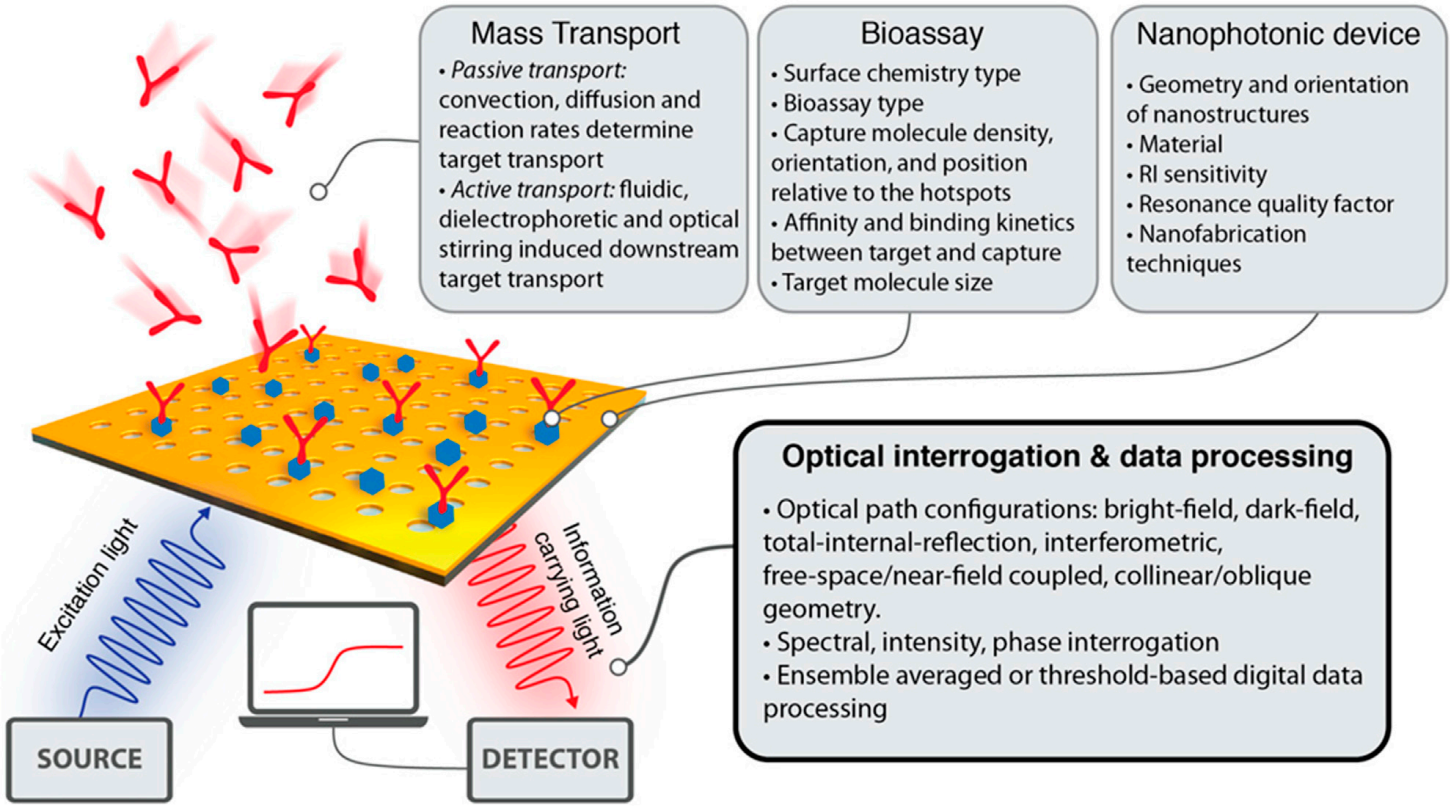
An outline highlighting the important variables such as biochemical, computational, and physical variables that affect the capability of nanophotonic biosensors to visualize the data (Yesilkoy, 2019).

Conversely, nanophotonic structures, on their surface, might firmly limit the light to a distance lower than 10 nm and produce the peaks of strong electromagnetic fields by mimicking a photonic nanostructure antenna. The strength of locally adapted fields can also be increased by more than 3–4 orders of magnitude of incident light by nanophotonic structures. With this strong field of localization, nanophotonic biosensors exhibit less sensitivity to background intrusions (Ngo et al., 2016; Galarreta et al., 2013; Cambiasso et al., 2018). Some areas are at their strongest and are spatially overlapping. These are the areas where the molecules of analytes are confined to the surface and may include areas where the evanescent field strength is substantial. Nanostructure resonance quality determines how proficiently these biosensors operate (Soler et al., 2020; Hayashi et al., 1988). The design of nanophotonic sensors provides very important attributes like total measurement time, a dynamic range, a detection limit, and directly connecting the numbers to the practicality of the light-based devices. Nevertheless, various important elements affect how efficiently the platform of the nanophotonic sensor executes in bioanalytics (see Figure 5) (Yesilkoy, 2019).

### 2.2 Plasmonic nanostructures-based biosensors

**FIGURE 6:**
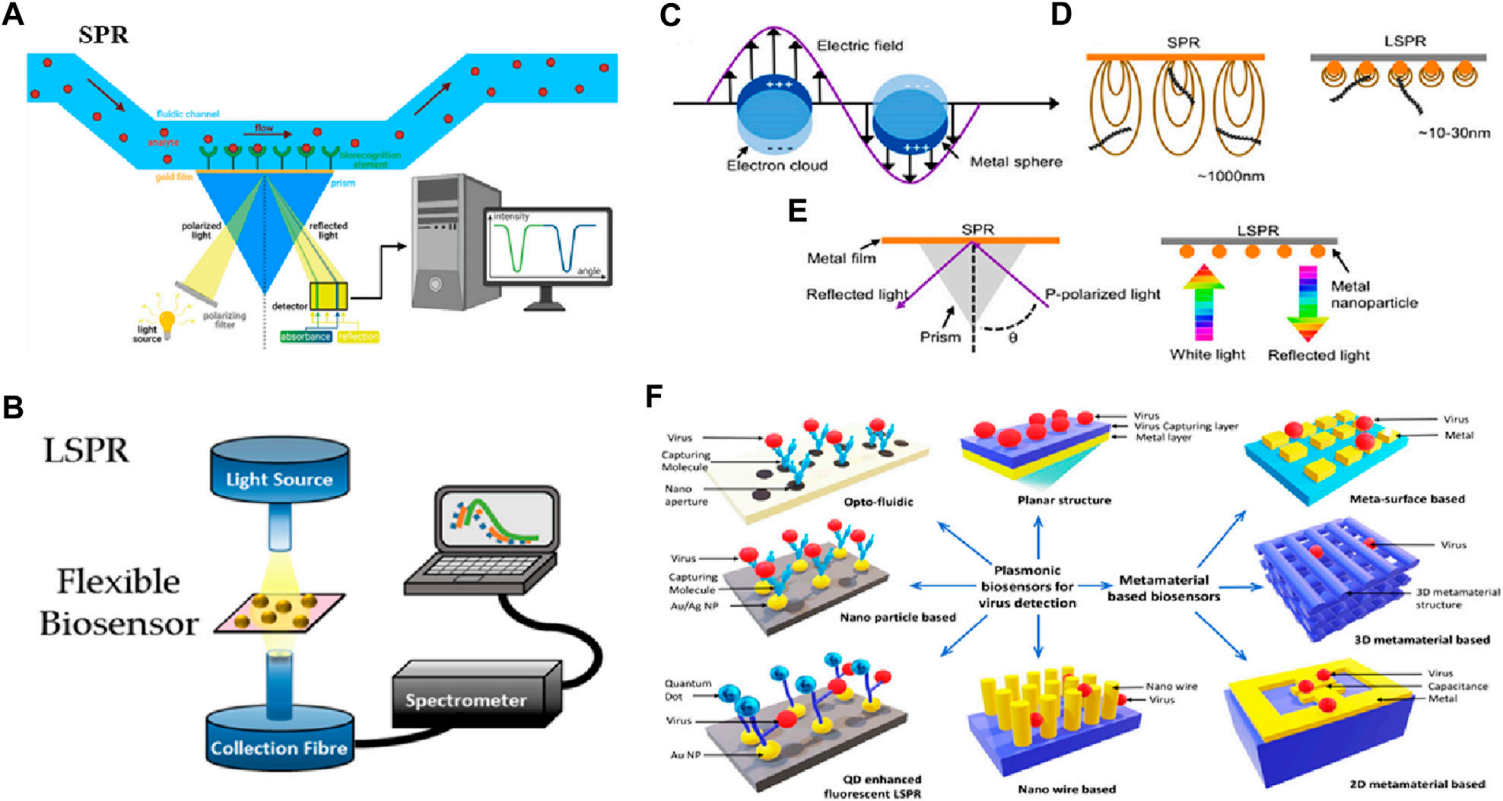
Biosensing based on SPR and LSPR. **(A)** SPR setup (Anand et al., 2022), **(B)** LSPR setup (Miranda et al., 2021), **(C)** LSPR from metal NP, **(D)** Comparison of SPR and LSPR, **(E)** Plasmon resonance criterion (Xiao et al., 2023) and **(F)** Various structures based on plasmonics and metamaterials used to probe biosensing (Hassan et al., 2021).

The early advances in nanophotonic biosensors are mainly focused on utilizing the distinctive optical properties of nanomaterials, for instance, plasmonic and photonic crystals, and facilitating the label-free and real-time observation of biological interactions. Exploiting phenomena such as SPR and gallery modes to disclose the little changes in the RI allows for the detection of the interaction of biomolecules at the level of a single molecule, with indications for clinical diagnostics. Recent advances including the incorporation of metamaterials in fabrication techniques such as nanoimprint lithography, have allowed for the development of low-cost, portable, and compact biosensing devices. As a substitute for conventionally propagating surface plasmon (PSP)-based nanoplasmonic and SPR biosensors, thin film, which is called plasmon-assisted surface metal nanostructures, became evident in the early 2000s. Figure 6A illustrates the typical setup of SPR used in biosensing (Anand et al., 2022). On the other hand, LSPs, which are abbreviated as localized surface plasmons, serve as the substructure for the foundation of nano-plasmonic biosensors and occur when light is limited to a small portion of the metal nanoparticle (NP) surface (see Figure 6C). In most localized surface plasmon resonance (LSPR) optical setups, an optical fiber probe is directly connected to a white light source. If the tool is optically transparent, the resonant spectra may be acquired in transmission mode; otherwise, devices with high reflectivity can be collected in reflectance mode. The transmitted and reflected light are collected using a spectrometer (see Figure 6B). The evanescent field of SPR is substantially longer than that of LSPR. It demonstrates that the micron perception range of SPR is far greater than the few tens of nanometers that LSPR can detect. Due to SPR’s large detection range, biomolecules that are not bound to the sensor surface may be detected in biosensing, resulting in false positives (see Figure 6D). Secondly, light momentum needs to be raised by adaptive optics since SPR finds it difficult to meet the plasmon resonance criterion (see Figure 6E). A summary is displayed in Figure 6, illustrating the detection mechanisms of SPR and LSPR setups, the differences between SPR and LSPR, and various structures based on plasmonics and metamaterials used to probe biosensing (see Figure 6F) (Miranda et al., 2021; Xiao et al., 2023). Plasmonic biosensing may have an optofluidic, quantum dot (QD), or NP-based structure, while metamaterial-based biosensors can be categorized as 2D, 3D metamaterial, or metasurface biosensors (Wang et al., 2023).

**FIGURE 7:**
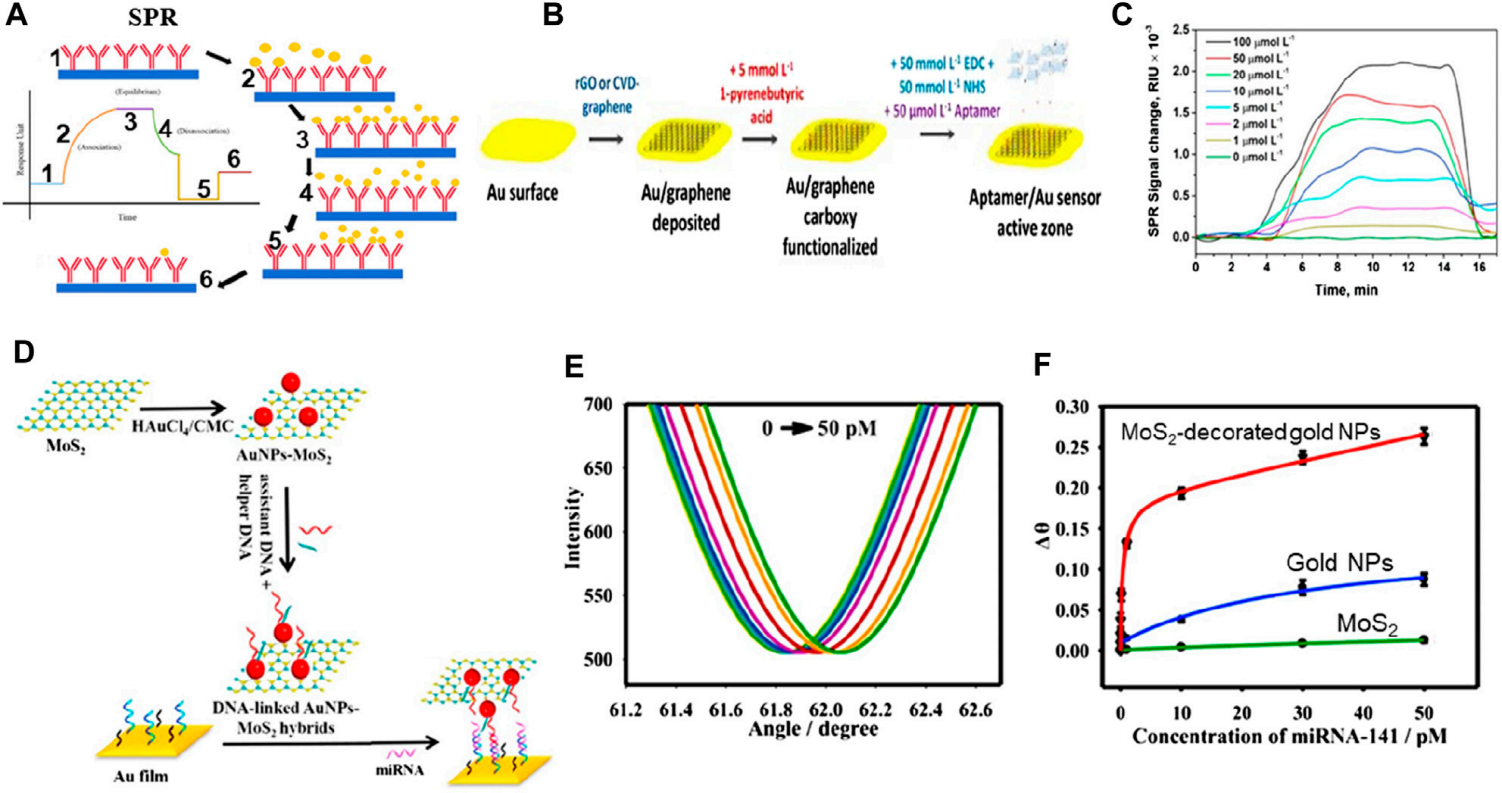
**(A)** SPR biosensing mechanism (Hassan et al., 2021). **(B)** Schematic of graphene-based biosensing setup, and **(C)** SPR signal (Écija-Arenas et al., 2021). **(D)** Schematic of MoS_2_-based hybrid materials for biosensing, **(E)** Change in reflectance as a function of angle at varying concentrations, and **(F)** Change angle as a function of varying concentrations (Nie et al., 2017).

SPR biosensors can also be optical biosensors operating on the principles of linear optics. The general method for analyzing biomolecule reactions involves immobilizing one interacting molecule on the sensor chip’s surface and continuously injecting the binding counterpart, or sample analyte, into the solution via the flow cell, causing the analyte to flow over the capturing surface. The analyte accumulates on the surface after engaging with the binding molecule, increasing the RI. The RI change is depicted in real-time, resulting in a reaction unit against a time plot. This mechanism is comprehensively shown in Figure 7A (Hassan et al., 2021). Owing to the material’s conductivity, SPR oscillations are extremely sensitive to changes in the material’s interface and can be potentially used in biosensing applications. Additionally, SPR-based biosensors are commonly used to measure fluctuations in the material’s index of refractivity. The generation of plasmons is caused by incoming light reflecting at an angle of SPR when the sensors are exposed. The field vector of this excited plasmon is sensitive to environmental variations caused by biomedical adsorptions; thus, when biomedical binds to the sensor, the RI of the sensor surface changes, triggering changes in the SPR angle due to the change in the electric field vector of plasmons.

Incorporating 2D materials with metals has been proposed as a viable method to enhance the performance of plasmonic biosensors owing to their unique characteristics, including strong charge transfer capabilities and a high surface-to-volume ratio (see Figure 7). Taking advantage of these important characteristics and incorporating noble metals, a chemical vapor deposition method was utilized for depositing a monolayer of graphene on the gold (Au) nanosheets to analyze biosensing outcomes (see Figure 7B). A linear concentration range of 0–100 μM was detected by this biosensor, with a limit of detection of 285 nM (see Figure 7C) (Écija-Arenas et al., 2021). There is still more research needs to be done regarding the mass manufacturing of graphene-based biosensors, particularly concerning fabrication efficiency (Nurrohman et al., 2020). Another study (Nie et al., 2017) reported good biosensing detection outcomes of 0.5 fM based on nanophotonic MoS_2_-decorated Au NPs (see Figures 7D–F). They reported that this outcome can be attributed to MoS_2_’s greater surface area, which allows for the attachment of more Au NPs in Au-NP-decorated MoS_2_ exhibiting a greater biosensing response when compared to Au NPs. Scientists have revealed that the mass transport and optical properties of plasmonic nanostructures influence the performance of biosensors, and customized nanostructures can determine the concentration of analytes 10 times less than the standard SPR biosensor can. The diagnostic process in the field of medicine is still considered a compound process for the scientific setup, irrespective of the advancements and advantages of research, civilization, and technology.

### 2.3 Resonant dielectric nanostructures-based biosensors

**FIGURE 8:**
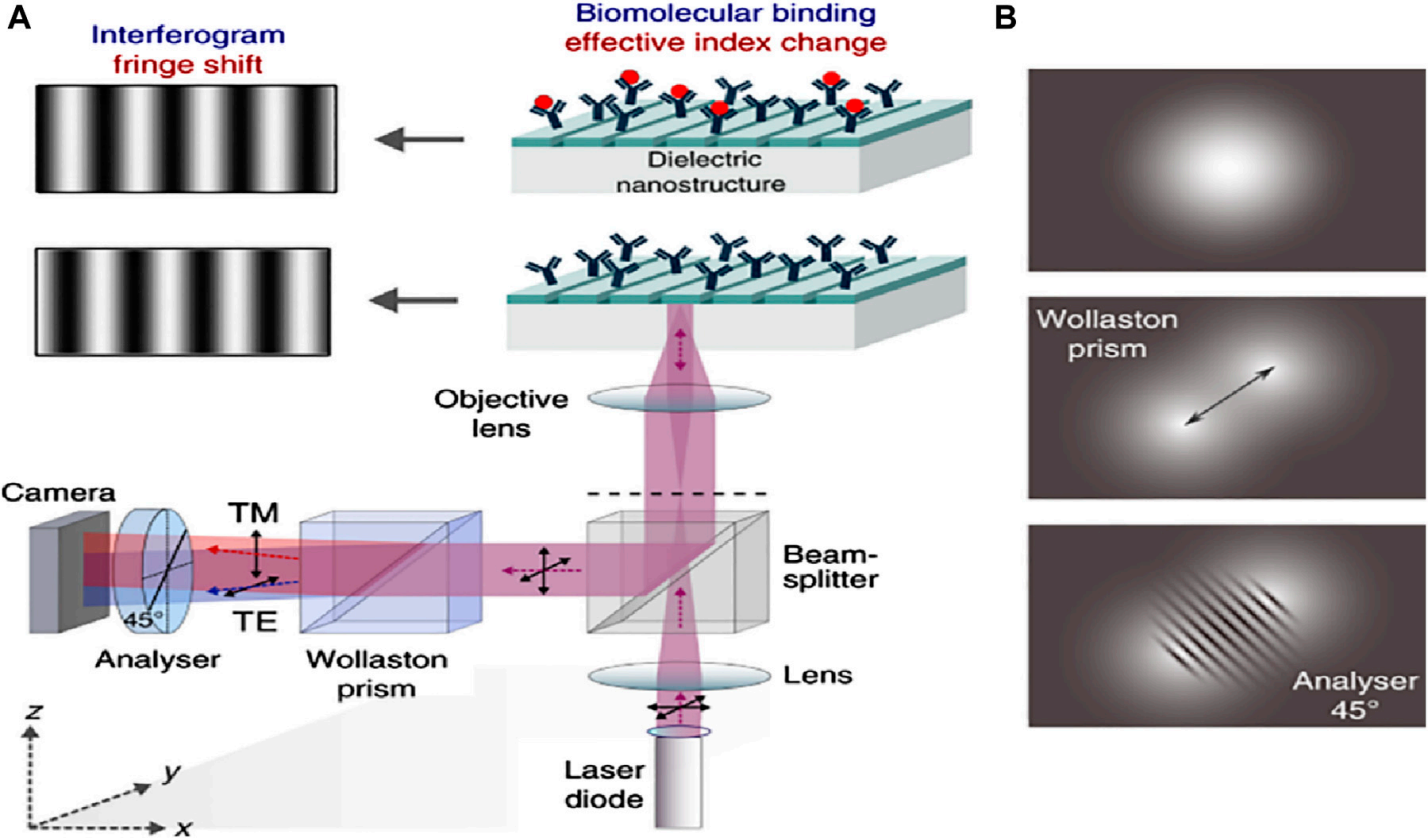
**(A)** Schematics of the optical setup of the phase-sensitive detection apparatus. The back focal plane of a 5-times objective gets a focused beam from an 855 nm laser diode. This setup confirms that the guided-mode resonance is stimulated by collimated light. In the grating grooves, the incoming light’s polarization is positioned at a 45° angle, making the TE and TM modes equally excitable. The sensor’s resonantly reflected light comprises phase information and the effective RI of the orthogonally polarized guided modes. With the help of the Wollaston prism, an angle of 1° is established within the two modes with an orientation of about 45°. The two beams bisect in the camera plane, building a highly diverse interferogram, and **(B)** At an analyzer orientation of around 45°, the two divergent beams meet in the camera plane, resulting in a high-contrast interferogram (Barth et al., 2020).

Plasmonic nanostructures derived from coinage metals have a very restricted array of spectral operations, for example, clear expansion of the plasmon-resonant field, in addition to loss and unreliability at elevated temperatures (Verma et al., 2022). Certain metals are being comprehensively investigated as the reciprocal of plasmonic components, including metal nitrides, doped oxides, metal sulfur compounds, and transparent conductive oxides. These can provide a distinct setup for area-amplified biosensing spectroscopy (Iqbal et al.). Procedures such as surface-amplified fluorescence methods are very effective due to the smooth effects of trivial dielectrics. Figure 8 shows the phase-sensitive dielectric nanostructures-based biosensing detection setup and its mechanism, in which a light-based system where the 5-objective’s focal plane was concentrated with the help of an 855 nm laser diode. This will make certain that resonance is elevated with the collimated light. As the polarization of incident light is 45° from the grating channel, the TM and TE modes become excited. The sensor’s resonantly reflected light comprises phase information and the effective RI of the orthogonally polarized guided modes (see Figure 8A). By employing the Wollaston prism, Isabel and his co-workers reported that an angle shift of 1° was established within the two modes with an orientation of about 45°, and the two beams bisect in the camera plane, building a highly diverse interferogram (Barth et al., 2020). With the help of the analyzer, the interferogram was collected in the overlapping segment of the beam, located in the camera, providing both beams with an identical state of polarization (see Figure 8B).

**FIGURE 9:**
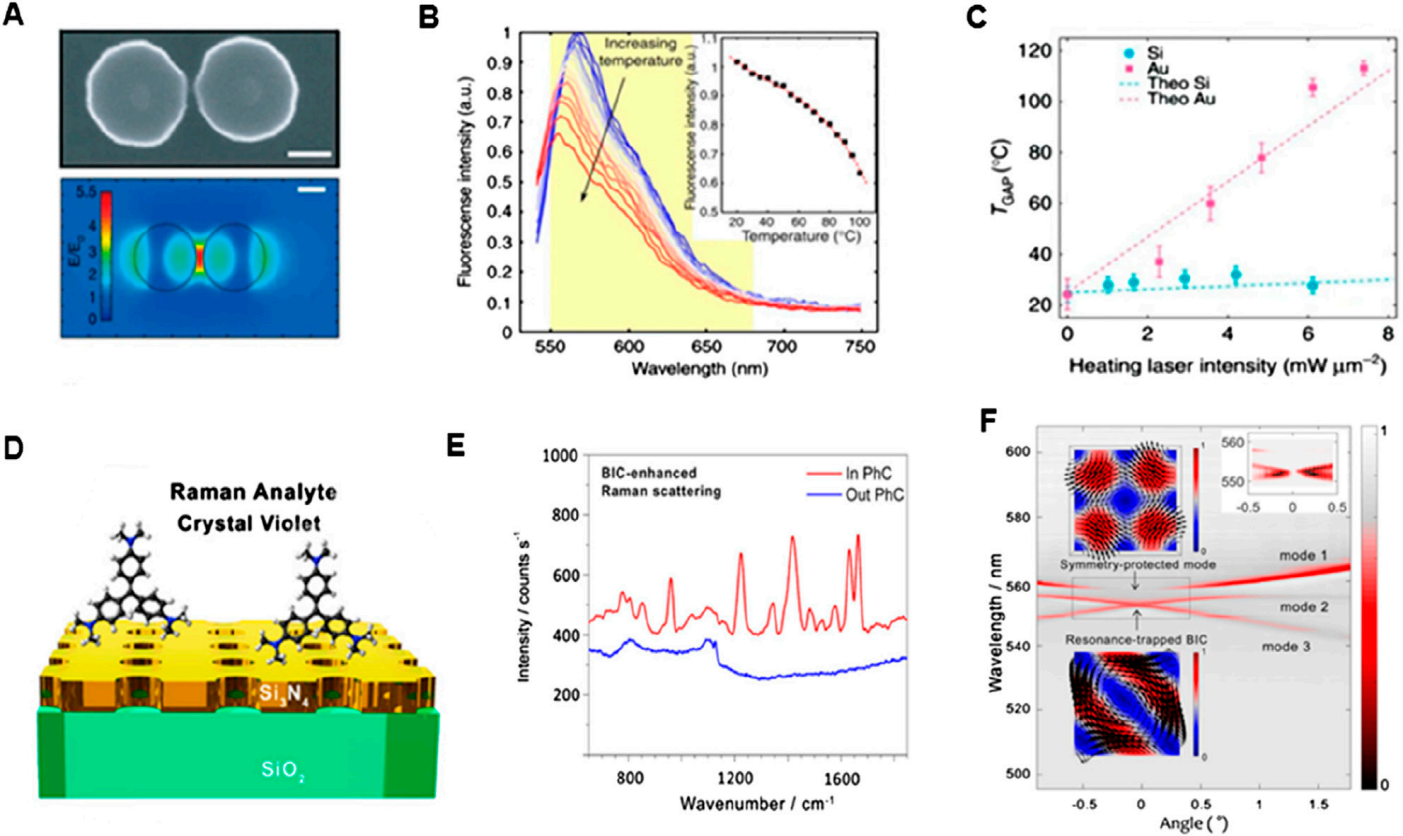
SERS-based biosensing **(A)** Incorporating the metasurface of Si-dimer nanoantenna, **(B)** Nile red emission spectrum as a function of wavelength. The inset shows temperature-dependent spectra and **(C)** Plots of temperature vs. laser intensity for Au and Si (Caldarola et al., 2015) **(D)** Schematic setup of Si_3_N_4_ exhibiting BIC mode, **(E)** Raman emission spectra of the CV in and out PhCM, and **(F)** Transmission spectra depicting dispersion bands (Romano et al., 2018b).

Dielectric metasurfaces have recently been presented as an alternative to their plasmonic counterparts in Raman spectroscopy. The high RI results in substantial light confinement and the materials used in dielectric metasurfaces have very low absorption losses, allowing for a reduction in heat generation. Caldarola et al. (2015) discovered that a slight temperature increase in the hot patches and surrounding areas resulted in inducing a significant amount of surface-enhanced Raman scattering and fluorescence when effective nanoantennas with low light-to-heat conversion rates were employed. These nanoantennas were produced using a specialized nanophotonic platform based on dielectric nanostructures (see Figure 9). The top area in Figure 9A depicts the size, height, and form of a single Si-dimer nanoantenna with two disks separated by 20 nm. This optical antenna performance around a Si dimer triggered the 860 nm resonance wavelength as shown in Figure 9A (bottom) along with the predicted near-field distribution. The structure’s dipolar resonance causes the strongest field amplification in the gap, yielding values of the field enhancement E/E_0_ close to 5.5 or so in this case, where E_0_ is the electric-field amplitude of incident light and E is the amplitude of the enhanced electric field. On the other hand, a theoretical estimation of maximum field increment for Si dimers yields E/E_0_ = 32 while keeping the gap as low as 4 nm (Albella et al., 2013), indicating that single-molecule surface enhanced Raman scattering (SERS) detection is possible (Le Ru and Etchegoin, 2012). The temperature properties of nanoantennas were examined by Caldarola et al. using a thermal imaging approach that combines molecular thermometry with a diffraction-limited spatial resolution, attaining a resolution of around 370 nm. The Nile Red fluorescence profile can be used as a probe to identify local temperature shifts because of its extreme sensitivity to temperature changes (see Figure 9B). As anticipated, the Au nanoantennas exhibit a temperature increase in the gap of more than 80°C, while the Si nanoantennas have a relatively low-temperature rise. The observed gap temperatures and those acquired from numerical simulations (dashed lines) show good agreement, which is consistent with theoretical views. Additionally, a heating slope ratio (Au/Si) of 17.6 was ascertained by the authors as shown in Figure 9C. Subsequent computational assessments of the temperature increase for heating laser intensities up to 1.2 × 10^2^ mW/m^2^ revealed that the gap temperature for Au nanoantennas would rise over 1,200°C, but only by 100°C for Si nanoantennas.

To increase the SERS signal amplification capabilities in this context (Romano et al., 2018a), optical bound states in the continuum (BIC) in the all-dielectric metasurface with silicon nitride (Si_3_N_4_) nanopores was investigated, and it was observed that the Raman signal of crystal violet (CV) dye molecules increased 103-fold with this metasurface (see Figure 9D). The selection of this CV dye was based on the dissimilarity of its Raman fingerprint and fluorescence emission spectra. As a result, unlike the Raman spectrum of R6G, the Raman scattering signal of CV does not fully integrate into the background of strong fluorescence. To boost signal amplification for resonance matching, the researchers slightly angled the sample angle by around one degree. Figure 9E shows the Raman spectrum on the photonic crystal metasurface (PhCM) driven by BIC enhancement. There was no visible Raman scattering in the unpatterned Si_3_N_4_. The TE band of transmission is shown in Figure 9F at a PhCM of 54 nm-thickness, exhibiting three bands of dispersion (1-3 modes). In addition, when a mode arises at a certain angle, the transmission experiences a very slight dip that eventually vanishes when the mode approaches the diverging Q-factor BIC mode (Hsu et al., 2013; Kodigala et al., 2017). The first mode, which is the symmetry-protected mode and must have an arbitrarily high Q-factor at 0°, represents a single degenerate mode. When moving away from the average occurrence (0° or, in other words, Γ point known as the origin in reciprocal lattice), its Q-factor significantly decreases (Romano et al., 2018b). The expected symmetry-protected BIC is shown in the top inset in Figure 9F. Though early efforts toward dielectric metasurfaces for SERS were made, metal-based antennas still provide superior SERS signals and greater field enhancements. Refinement of the meta-atom designs that permit high-Q resonances and the maximum near-field improvements outside the resonator volume, however, can lead to even better dielectric metasurfaces. As a result, biomolecules are more likely to reach near fields, resulting in a stronger SERS signal.

### 2.4 Surface-enhanced spectroscopies-based biosensors

**FIGURE 10:**
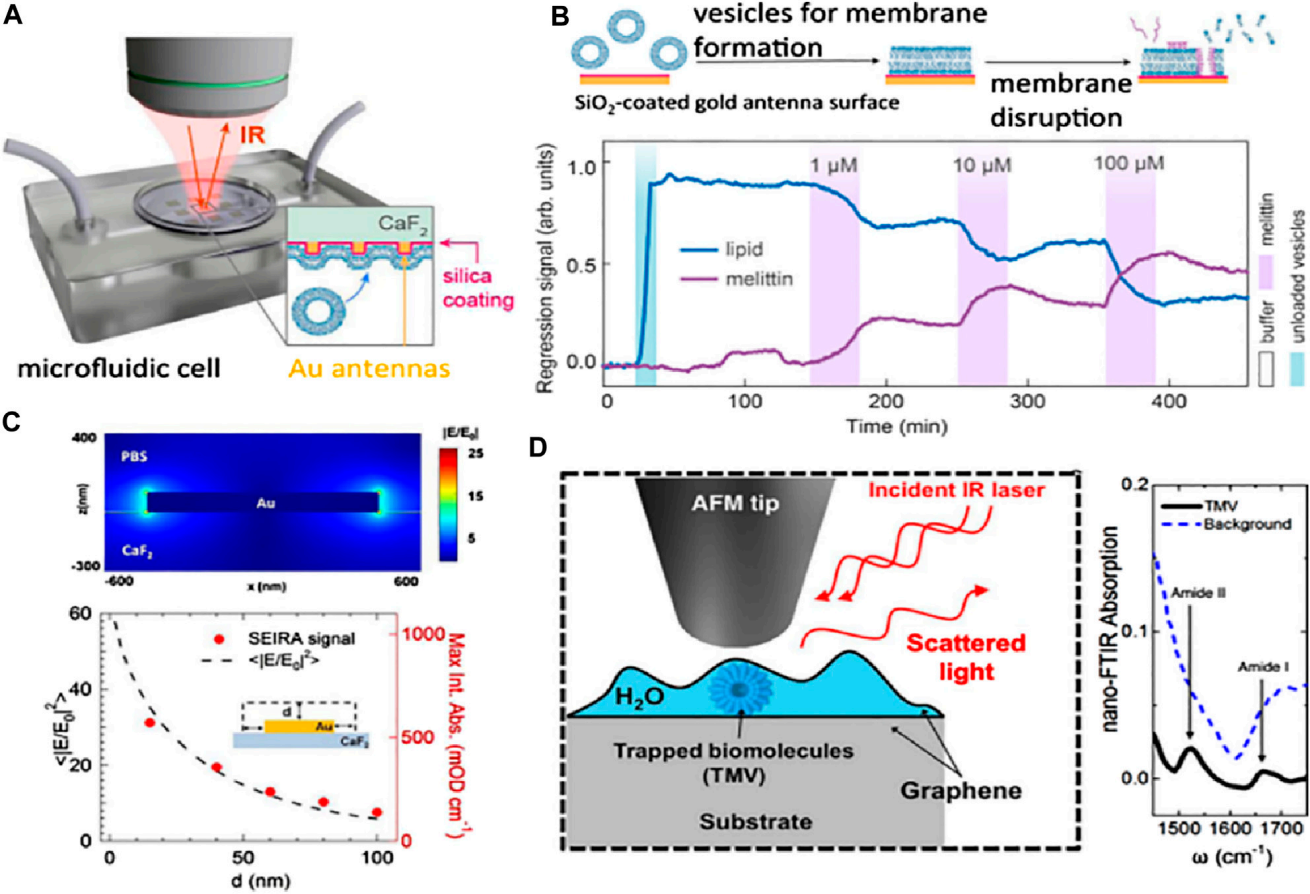
**(A)** Schematic setup for the phase detection of analytes *in situ* SEIRA based on reflection mode. **(B)** The lipid membrane has been monitored with the help of molecular-specific *in situ* SEIRA over the Au antenna coated with SiO_2_, where the bursting of lipid vesicles occurs with the help of cytolytic peptide melittin injection, resulting in the formation of a lipid bilayer membrane. **(C)** Buffer containing Au nano-antenna, and the electric-field outcome. The signal dependence of SEIRA has been displayed after contrasting it with computer calculations (shown as dots) and absorbance data. **(D)** Nano FTIR absorption and setup of a graphene liquid cell depicting the mechanism of trapping biomolecules in the water layer (Oh et al., 2021).

The aspects influencing the accuracy of dielectric affinity biosensors and nanostructured metals for all applicable analytes are the characteristics of the receptors, which might not always be accessible (Liedberg et al., 1995). There is a possible augmentation of refractometric biosensors with vibrational spectroscopies (Baker et al., 2016), and distinct techniques can demand Raman scattering and infrared absorption, which can supply the analyte selectivity without providing details about the molecular structure of the analyte and the receptor. These methods induce the resonance frequency in the molecular vibrations, influencing both the chemical makeup and bond conformation. Consequently, these can be used as an alternative to observe the molecular fingerprints, and in absence of external labels, they can be used to inquire about the molecular conformations. The primary hurdle in utilizing the low-volume biological specimens treated with vibrational spectroscopy is their relatively low sensitivity. For sonification analysis, the weak signal can also limit the approach of the vibrational spectroscopy method. The relatively considerable mid-range infrared light assimilated by the water may conceal the signal of the analyte and provide a new hindrance for the biosensors in the aquatic environment. By employing surface-enhancing procedures such as SERS and incorporating nanophotonic materials, one can refine the analyte signal outputs (Baker et al., 2016; Ataka and Heberle, 2007; Langer et al., 2019; Neubrech et al., 2017; Mudumba et al., 2017). Figure 10 shows research applying resonant Au nanorods shielded with a SiO_2_ thin layer to foster biomembrane formation, considering an exceptional elaboration of these advantages. SiO_2_ layering helps in characterizing the surface-enhanced infrared absorption (SEIRA) and enhancement factor (EF) distance dependency. Sang-Hyun et al. (Oh et al., 2021), showed that the assimilated signals from the molecules can be detected for overlayer thicknesses of SiO_2_, hence indicating the separation distance of metal from the molecule to the limit of 100 nm in fluidic conditions (see Figures 10A–D). This relationship of distance dependency completely aligns with the Au antennas for the characterization of dry SEIRAs. The SEIRA is widely used as it allows the receptor molecules to be used for selectivity and also helps in observing the larger analytes. On the other hand, the SERS has very low probing ranges of about a few nm for objects like extracellular vesicles and biomacromolecules. Through literature studies, it has been revealed that graphene catches sample solutions with a small layer in aqueous conditions for tip-enhanced IR spectroscopy (Oh et al., 2021).

**FIGURE 11:**
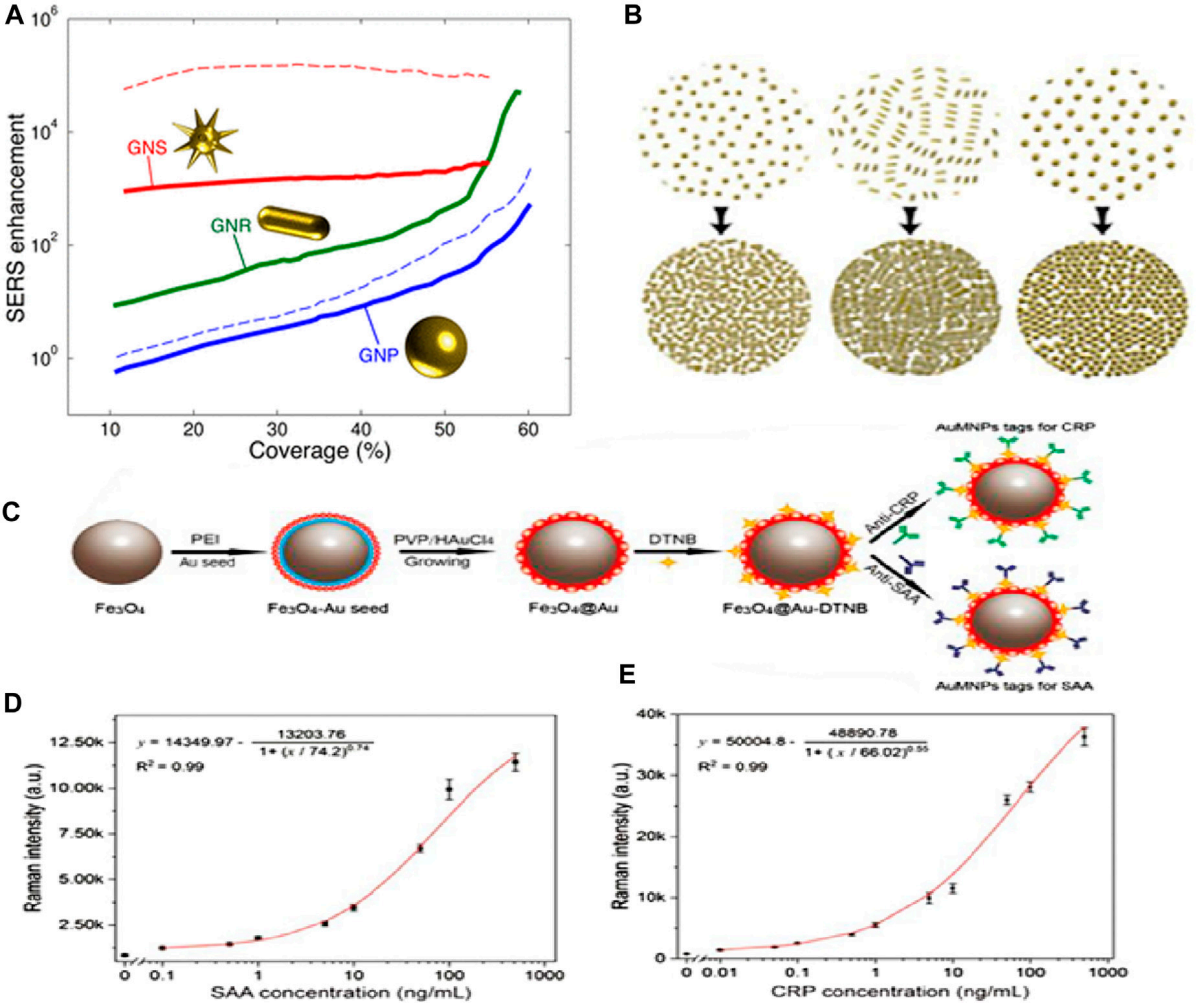
**(A)** SERS improvement is based on coverage. At 785 nm, the incident light is shown as solid lines. The dotted lines represent incident light at 633 nm (blue) and 900 nm (red) for nanospheres and nanostars, respectively, and **(B)** This schematic depicts the monolayers’ increased coverage (Solís et al., 2017). **(C)** The manufacturing scheme for antibody-conjugated Fe_3_O_4_@Au SERS nanotags, **(D)** SAA and Raman intensity calibration curves, and **(E)** Raman intensity and CRP calibration curve (Liu et al., 2020).

The SERS signal intensity is mostly influenced by the interfacial interactions that occur between the molecules under study and the plasmonic nanostructure. Thus, the biosensing sensitivity is primarily determined by two critical parameters: the highest number of hot spots and the improved interaction of the target molecule with effective hot spots. Nanostructures’ sharp edges can significantly boost the intensity of Raman scattering. Solis et al. (Solís et al., 2017) demonstrated that Au nanostars outperform nanospheres and nanorods in terms of SERS enhancement (see Figure 11A). Conversely, the morphologies of NPs, such as nanostars, that offer significant electromagnetic improvement at the single-particle level do not always improve in tightly aligned arrays. On the other hand, simpler geometries such as rods and spheres can greatly boost SERS enhancement when their surface density becomes close to full coverage (see Figure 11B). Interestingly, it was observed that plasmon-coupled nanorods, whose surface coverage is greater than 60%, performed better than decoupled nanostars. Nanorods were interesting candidates for synthesizing efficient SERS substrates since they outperformed nanosphere arrays by two orders of magnitude and performed comparably, if not better, than nanostars. The shape of the nanoparticles can be tuned to deliver the optimal electromagnetic boost for each excitation wavelength and Raman shift. In another study, Liu and his team (Liu et al., 2020) reported the formation of SERS functionalization of Fe_3_O_4_@Au magnetic NPs by including a lateral flow immunoassay strip. This strip aimed to analyze dual infection indicators simultaneously and with ultrasensitivity (see Figure 11C). Antibody-modified Fe_3_O_4_@Au magnetic NPs were used for constructing the SERS nanotags and effective enriching tools that quickly identified and captured the target infection indicators from blood samples. C-reactive protein (CRP) and serum amyloid A (SAA) were shown to have LODs of 0.01 ng/mL and 0.1 ng/mL, respectively (see Figures 11D, E). These magnetic SERS strips exhibited exceptional specificity, stability, and selectivity when examining complex materials and showed great potential for application in the identification of infectious illnesses.

### 2.5 Phase-driven nanophotonic biosensors

A continuous investigation is in progress to increase the reactivity of nanophotonic biosensors, specifically after utilizing the process that is more closely related to photonic resonance. The main objective along this line is to facilitate label-free biosensing with the maximum resolution of the RI while decreasing the specification in nanofabrication. In the past years, effective detection techniques have been implemented that support spectral or angular data combined with intensity-based strategies as transducers in the domain of non-label biomolecular assays with the help of nanophotonic biosensors. These innovations have resulted in the evolution of different systems that display competitive reactivity or, in some instances, are better than the standard diagnostic enzyme-linked immunosorbent assay (ELISA) (Gan and Patel, 2013). Taking into account the steep phase method, new strategies will be used to attain enhanced sensitivity in basic systems. There are two parallel paths, direct-phase interrogation, and indirect-phase detection, examined for phase-driven growth. In the direct-phase interrogation method, it utilizes interferometry directly to investigate the photonic resonances. This method has shown an increase in sensitivity in both dielectric and plasmonic systems. Besides this, the other method is indirect detection that harnesses phase phenomena, for example, advanced intensity-dependent detection.

**FIGURE 12:**
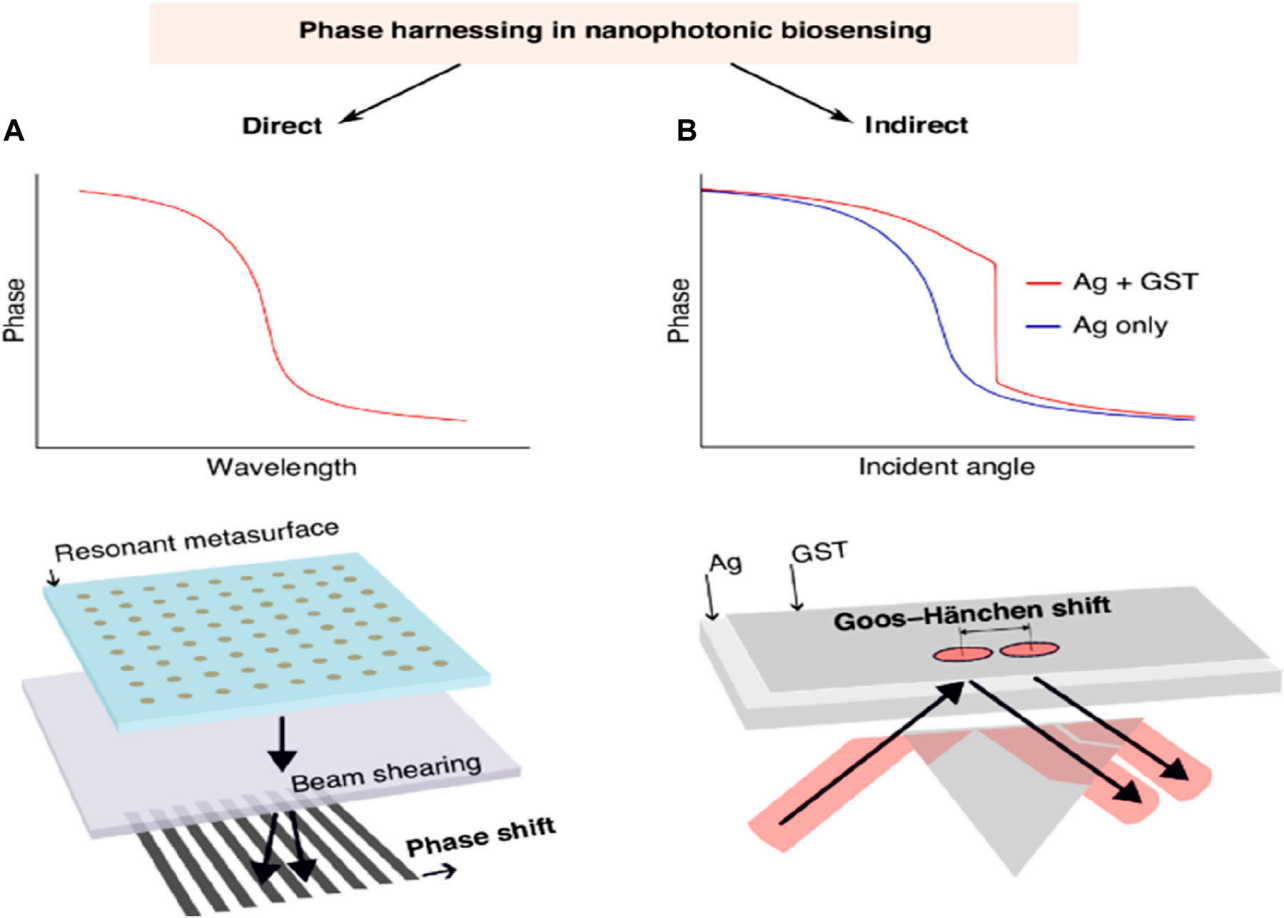
**(A)** A diagrammatic depiction of phase behavior and direct phase interrogation via polarization beam shearing, inspired by an interferometric dielectric platform (Barth et al., 2020) and a phase-sensitive plasmonic biosensor (Yesilkoy et al., 2018), and **(B)** Schematic represented by Zhu et al. (Zhu et al., 2024). This photograph is adapted from (Barth and Lee, 2024).

A lot of attention has recently been paid to magnified Goos-Hänchen (G-H) shifts and topologically protected phase singularities in biosensing (Barth and Lee, 2024). Zhu et al. have made noteworthy advancements in the study of G-H shift-based sensing. In particular, their approach produced singularized phase responses by integrating an atomically thin Ge_2_Sb_2_Te_5_ (GST) layer on top of a silver nanofilm. G-H shifts are tiny lateral beam displacements brought about by phase-shifted beam components interfering with each other and total internal reflection. As a result, the shift depends on the RI at the interface because of evanescent waves. A single-layer dielectric interface may produce modest beam displacements of hundreds of microns due to steeper phase responses caused by resonances and greater absorption with additional materials. This is because the G-H shift is proportional to the phase shift discrepancies observed during complete internal reflection. Their results show a steep phase curve (see Figure 12A, B) and significant position changes of up to 440 μm, surpassing earlier achievements with comparable approaches (Zhu et al., 2024). The phase singularity linked with the planned absorption in the GST layer is responsible for these results. They evaluated both the sensitivity and the noise of their system, demonstrating a LOD of 7 × 10^−7^ RIU. This result is close to several direct phase-sensitive SPR modalities (resolution 10^–8^ RIU) and superior to other more straightforward direct phase interrogation techniques (LOD down to 10^–6^ RIU). The stated results in phase-sensitive modalities should be acknowledged to have no validity in the previously determined LOD and its relationship to system noise. This noise can significantly exceed the theoretical phase resolution in an unstabilized biosensing system that experiences both mechanical and temperature drift. Their research showed that small cytokine biomarkers could be detected label-free at concentrations as low as 0.1 fM, which is a major advancement over earlier techniques. However, further validation of these proof-of-principle detection limits would necessitate testing in complex matrices with adequate biochemical controls, including serum (Barth et al., 2020; Barth and Lee, 2024; Yesilkoy et al., 2018).

## 3 Future outlooks and challenges

**FIGURE 13:**
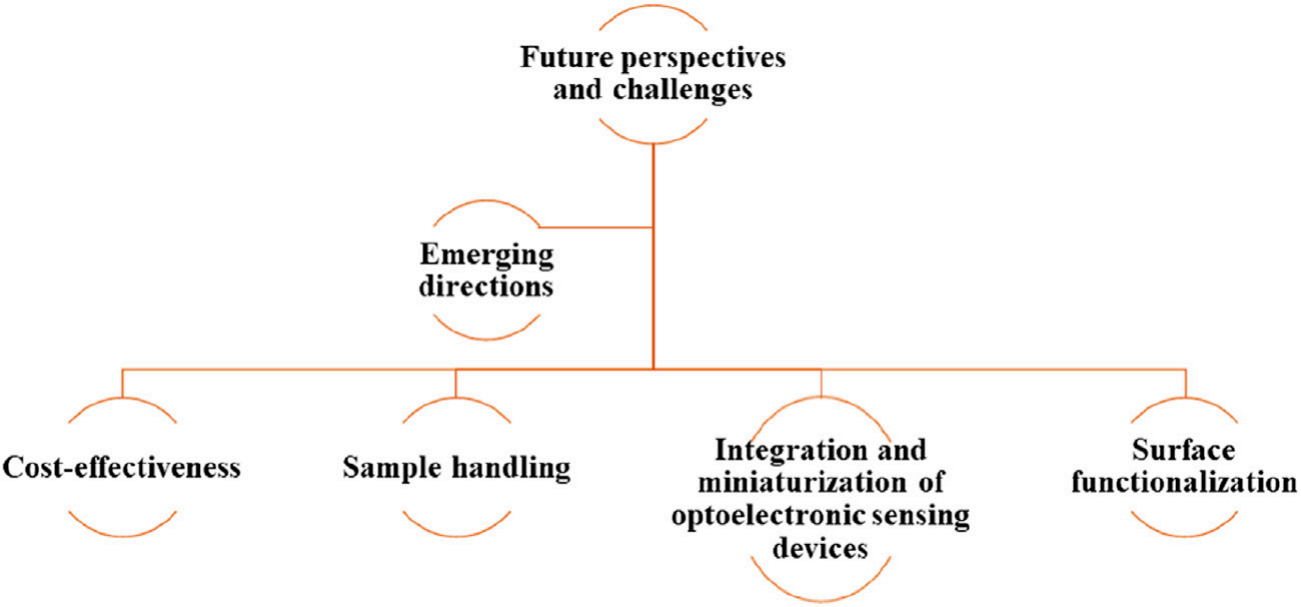
An illustrative overview of challenges and perspectives of nanophotonic biosensors.

Nanophotonic biosensors that can be used across a variety of biological fields ought to be upgraded, not only through research in the bio-photonics domain but through a wide interdisciplinary effort as well. Mentioned further is the overview of various scientific ultimatums and developmental potential platforms. An illustrative overview of the challenges and outlook for nanophotonics has been portrayed in Figure 13.

### 3.1 Integration and miniaturization of optoelectronic sensing devices

The developed nanophotonic biosensors offer much preferable probability, minimization, and output because of the strengthened capability of photonic integrated circuits composing them. The two methods that are involved in this are vertical integration and planar integration. During vertical integration, in contrast to the requirements for standard prisms, waveguides, or gratings that are visible light couplers, nanostructures can reduce the light coupling requirements. Certain factors, like lens-free phase, intensity, or hyperspectral imaging, have in recent times been displayed as the best approaches. Nanoscale structures that can be excited by resonance with incident light in a coherent optical pathway are beneficial for shrinking and multiplexing (Romano et al., 2018a; Nangare and Patil, 2020; Oliverio et al., 2017). Nanomaterials can also operate as transducers because of their sub-wavelength sensing zones, enhancing their productivity in nano-sized dimensions. This kind of situation could result in enormous multiplexing in theory, having only the physical restriction as the optical resolution light limit. Each of the nanostructures can also be implemented and operated in optics independently. Regardless of how, in the application of real-world sensing elements, separate sensing elements can also use the nanopattern arrays to create a greater signal than that of discrete particles. These elements can be arranged in 2D arrays and can also be used for detection with multiplexed analysis (Oliverio et al., 2017; Aksu et al., 2010; Wen et al., 2024). To transmit the light across any sample, vertical integration is needed, and one must reflect on the optical loss, scattering, and interferences during the analysis of vastly turbid and absorbent solutions. While employing the waveguide to escort the light, the best possible method to improve the performance of the sensor is to incorporate nanopatterns and sub-wavelength nanostructures (Huang et al., 2013; Escobedo et al., 2012). For instance, NPs have recently been developed over the wave ducts to synchronize the spectra and enhance their susceptibility to refractometric methods with continuous subwavelength confinement of the sensor volumes (Oliverio et al., 2017; Aksu et al., 2010).

**FIGURE 14:**
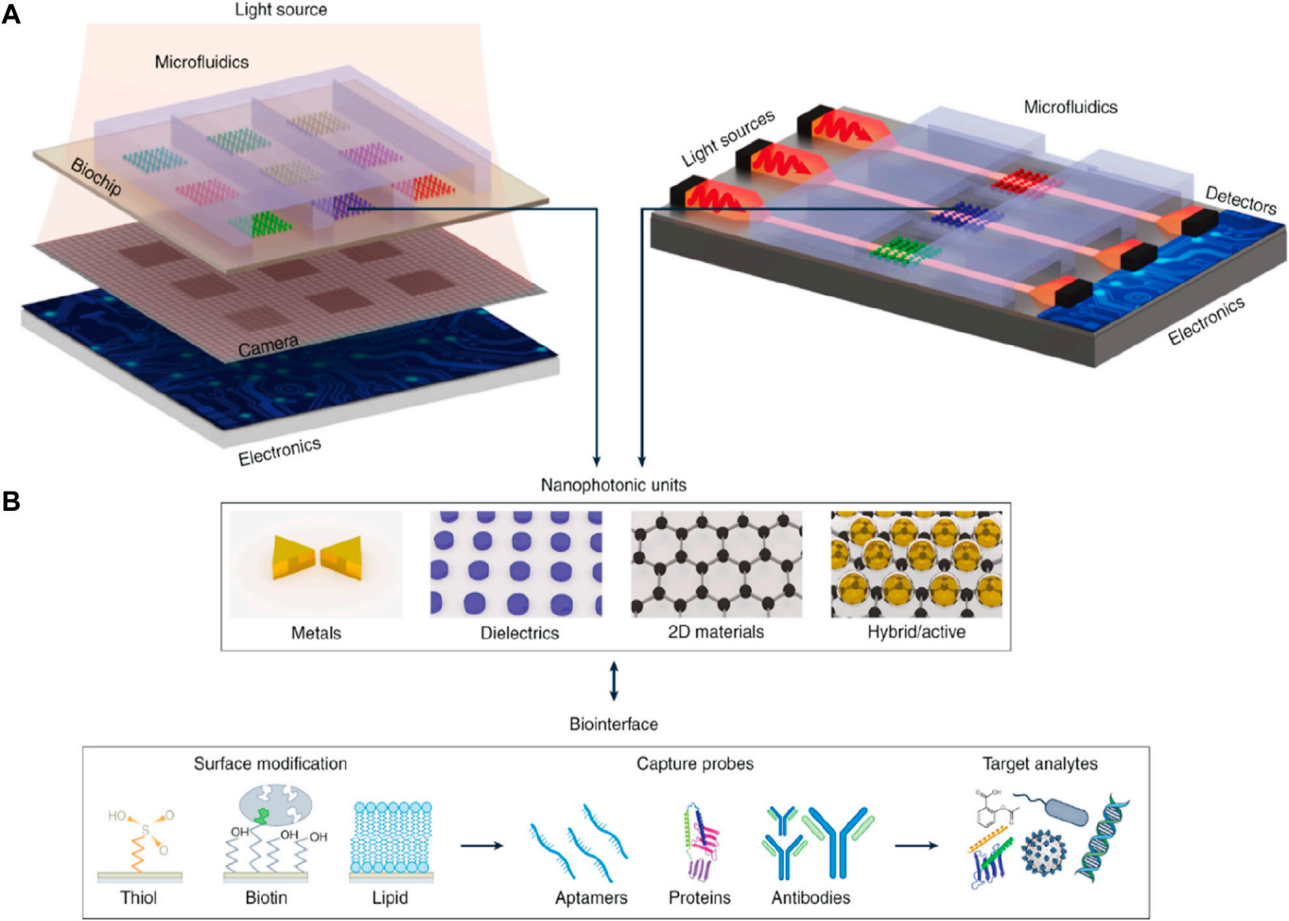
**(A)** Planar and vertical systems for integrating the biosensors, including several different layers and elements, and **(B)** Illustration of the nanophotonic components along with the most commonly used biofunctionalization surface methods (Altug et al., 2022).

There is an advancement in photonic biosensors due to enhancements in photonic integrated systems, and with the help of this advancement, there is an achievement in improving miniaturization, mobility, and performance. Integration has been classified as a vertical and planar system, as shown in Figure 14. In the case of a vertical integration system, nanostructures, as compared to traditional SPR, which in some cases requires exterior optical couplers, for instance, gratings or prisms, can facilitate light-coupling conditions. Exciting the resonant nanostructures along the passage of collinear optical light with the incident light is beneficial in attaining multiplexing and miniaturization. In recent times, this has been illustrated with the lens-free, phase-intensity hyperspectral imaging methods (Altug et al., 2022). The vertical integration method that functions in a transmission manner has been illustrated in Figure 14A. Here, the performance of the nanostructure can be expanded to nanoscale dimensions when the nanostructure, along with its subwavelength detection zone, can act as a transducer. Fundamentally, this possibility can lead to immense multiplexing if we take only optics into account and postulate that nanostructures can only be optimized specifically. The only limitation here could be the optical resolution limit. Practically, individual sensing components use the arrays of nanostructures to create a robust output signal as compared to discrete particles. These components can be organized into 2D patterns and modified for multiplexed detection. As the vertical integration system includes the passing of light across the sample, it is required to analyze the possible challenges of investigating the turbid samples because of interferences, optical loss, and scattering effects. The planar integration system includes the utilization of optical waveguides with the help of sensors, which can be organized in a 1D pattern and manipulated for multiplexed detection as shown in Figure 14B. The elevated level of miniaturization can be attained in this structure compared to that of a vertical system by combining the electronics layer over the surface of individual sensor chips, either homogeneously or with the help of hybrid bonding.

### 3.2 Cost-effectiveness

Economical biosensor chips, as well as single-use, have been reported to block impurities, demanding the cleaning process during the management of biological materials. As far as integration is concerned, the most useful alternatives are single-use cartridges and standalone readers. The photons need to be kept away from any source of light or detector while being accommodated within the cartridges. These cartridges can also be engineered to utilize the same reader to catch numerous samples. This layout decreases the costs of readers by permitting the utilization of economically accessible optoelectronic components. The other advantages include methods such as those related to the reproduction of the surface, which commonly decreases the total fetch and also enhances the sensor execution over time. To make use of low-cost cartridges, this technique must be used with alertness because of the high cost of biomaterials. Because of the efficient light source, nanophotonic biosensors and smartphones are progressively combined. Communication access, processing of pictures, and different camera tools can decrease costs and sanction extensive distribution (Yesilkoy et al., 2018; Ndukaife et al., 2016). With the help of customized apps, these sensors can also be used to examine the data, evaluate the signals from different samples of patients, and transfer the outcomes to the clinician wirelessly for interpretation. However, the more portable and with smaller footprints, the better the properties of planarly integrated biosensors, as their cost is more likely to be greater in a single-use plot because of the complex production methods needed to develop the numerous packaging and device layers. As different nanostructures can be figured differently, there is much research for producing nanophotonic biochips, primarily focused on the beams of ions or electron beam lithography. However, the lower bandwidth and higher cost of serial patterning methods have developed the requirement for alternate cost-effective production methods for retailing, using Moore’s law, which majorly indicates that making use of foundation and constructing practices congruent with silicon is one of the practical strategies (Maan et al., 2020; Hinman et al., 2018). A literature survey has shown the utilization of semiconductor foundries, irrespective of the early development of this field. In this case, the material possesses a significant hurdle: for example, front-end CMOS (Iqbal et al., 2023b) modification is contradictory to silver and gold. The research predicted that low-cost and large-scale lithography techniques would get additional attention as alternatives to various manufacturing techniques (Lísalová et al., 2017).

### 3.3 Sample handling

Technologies such as microfluidics can enable tasks such as the preparation of samples, sample focus, and transport of analytes; in the meantime, decreasing the necessary amount of samples and utilizing the reagents are beneficial for biosensor integration. However, nanophotonic biosensors can also be obtained from field use; lengthy detection times might occur from the elevated susceptibility confinement in the wavelength focal points and stagnant analyte transfer because of restrictions in the movement of mass from huge samples to the hot regions. At the moment, many approaches addressing this matter are being investigated, together with incorporating the microfluidic elements that enhance the transportation of analyte to the surface of the sensor and enable the concentration of analyte (Hu et al., 2019; Lee et al., 2019). There is another challenge that may include the electric field with the external lasers in detaching the microfluidic components like pumps and valves. To deliver the liquid samples, surface tension effects with the help of capillary microfluidics can be used without making use of any additional equipment. A new technique, digital microfluidics, is used to manipulate the microdroplets with the help of electric forces. In this vicinity, metallic nanostructures can work as both pumps and electrodes, allowing them to incorporate digital microfluidics in exploiting the picoliter-sized droplets without utilizing valves or pumps. In real-time biosensing, a critical factor is the process of collecting the sample. For instance, the matrix composition and broad range of analytes help in comparing the fluids utilized in medical diagnosis with the samples of foods utilized in food safety (Dahlin et al., 2012). This in-demand biofluid for the recognition of biomarkers is the blood. Even though blood withdrawal is the least intrusive, it still demands the segregation of blood components and restricts the effects of the matrix, which is generated by impediments like proteins, immunoglobulins, and cells. During the high concentration of analytes, a buffer is used for sample dilution and can lessen the matrix effects and help in detection; yet, an elevated concentration of the sample is required to recognize the rare analytes. There has been much research on biofluids such as sweat, saliva, or urine, and these samples can easily be accumulated without causing any trouble to the patient for analysis (Hentschel et al., 2017).

### 3.4 Surface functionalization

Non-target elements present within the matrix need to be treated as they move toward the surface of the biosensor in a non-specific way, which is quite a problem for complex clinical samples. The analyte with the lowest concentration, for example, is significantly inferior to those discovered in pathological samples, and they might be determined when there are no impeders like proteins, cells, or immunoglobulins (Malik et al., 2022b; Iqbal et al., 2024). To make the receptors immobilized effectively, implementation techniques that enable the complicated samples to become resistant to the non-specific bindings are mostly needed (Bauch et al., 2014). The traditional methods used for non-specific binding suppression include the use of polymers or protein inhibitors like bovine serum albumin or casein. Even though, in medical samples, these traditional methods recommend only a moderate immunity to non-specific binding, various approaches use zwitterionic polymers or copolymers, and they have now been inspected to inhibit the complicated culture medium from befouling. The certainty is that the anti-biofouling coatings in affinity biosensors frequently require an immobilized receptor, and these can be put on the outer side of the hot zone with a minimum diameter of about 10–20 nm, which is counted as an important element to take into consideration. The other characteristic to consider is the size of the receptor. Receptors of small size, such as nanobodies and aptamers, are anticipated to be utilized more often to guarantee the analyte’s binding to the receptor in hot regions. The two popular techniques for immobilization are covalent binding (using receptors like thiol, aldehyde, carboxyl, or epoxy) and streptavidin-biotin binding. Visser et al. and Lubken et al. have illustrated that molecular switches can allow transformable interactions in the spot of high-affinity receptors, frequently favored for stronger interactions (Cui et al., 2020; Wu et al., 2018a; Akkilic et al., 2020). Nanostructured biochips frequently contain a rugged sensitivity along the surface of the sensor because of the display and materials, contrary to traditional biochips having flat surfaces. These elements make implementation strategies complicated, demanding innovative techniques or methods for targeted functionalization of them. Zijlstra et al. explained the targeted method that allows the particular development of Au nanorod tips (Zijlstra et al., 2012). Galloway et al. utilized the absorption method of three photons for light-assisted modification to detect proteins present within the plasmonic dimer’s focal point (Galloway et al., 2013). Plasmonic tuning was used by Tijunelyte et al. to explain the confined click reaction near the Au NPs (Tijunelyte et al., 2016).

### 3.5 Emerging directions

Materials research has improved over time, and it has become evident that nanophotonic biosensors will continue to improve as an area of fundamental optics. With the help of 2D quantum, phase-change materials, and hybrid photonic materials, active and tunable reconfigurable biochips are made possible (Huang et al., 2020). One example is that, with the help of electrostatic gating, graphene’s distinct optoelectronic attributes can allow the robust control of plasmonic resonances (Malik et al., 2022c). This feature was used by Rodrigo et al. (Rodrigo et al., 2015) to advance mid-infrared plasmonic biosensors. Tight near-field constrictions were attained by utilizing the atomic layer thickness of 2D materials and acoustic graphene plasmons. These constrictions are beneficial for the identification of small molecules. Likewise, light production makes it feasible to generate small optically or electrically manageable biosensors without requiring an external light source by utilizing graphene, hybrid nanomaterials, or metals consisting of active elements such as quantum dots. Furthermore, a diversity of non-optical and optical methods used for detection on a single platform, like impedance, electrochemical, or mechanical platforms, may make it feasible to employ multipurpose biosensors to obtain more details from a given sample. SERS and SEIRA, refractometric sensing, can all be brought out on a similar platform utilizing nanophotonic biochips with a wide-ranging electromagnetic spectrum. Another method that can be used is chiral sensing, and currently, meta-surfaces are the attracting technique to generate the super-chiral fields for sensing and segregating the chiral molecules in biomolecules (Huang et al., 2020; Jung et al., 1998; Wu et al., 2018b; Lindquist et al., 2019). However, these studies, mainly centered on label-free techniques and surface-enhanced luminescence procedures like frequency upconversion, fluorescence, and chemiluminescence employing plasmonic and dielectric nanostructures, are also encouraging. Collecting more details to use artificial intelligence will completely expand the degrees of privilege in optics for temporal, spatial, spectral, and polarization. Future digital health services systems on the internet can integrate biosensors and autonomous operations to provide efficient healthcare services (Willets and Van Duyne, 2007; Limaj et al., 2016).

## 4 Summary and outlook

Nanophotonic biosensors have recently been significantly developed, leading them to exhibit adequate sensitivity to identify single molecular binding events. On the other hand, supplementary efforts to develop biosensor devices on multiple levels are necessary for further scientific advancements along this line. It is predicted that investigating nanoscale chemical and physical phenomena, which are pervasive with newly discovered outcomes, will promote the fabrication of much improved sensing devices suitable for next-generation bio-detection systems. Cost-effective, small-size photonic biosensors would be attainable with the help of well-established integrated technologies in the domain of nanophotonics and optoelectronics, which can be potentially linked to industrial facilities. In the course of the COVID-19 pandemic, which appeals for prompt, precise, and mobile diagnosis, the need for biosensors has become instantly apparent. Biosensors can be preferred over conventional testing devices, which are expensive and demand a proficient test operator, because of their simplicity, ease of detection, and capability to be tested at the location of the victim. Researchers are now in the process of designing more precise, more dependable, and more miniaturized biosensors.

An encouraging methodological approach for sensitivity enhancement in the future is the use of nanophotonic structures coated with advanced layers. The top layer of the coating will help catch the target analytes. This approach may elevate sensing capability even in the analysis of complex biological samples. Combining microfluidics and nanophotonic biochips will become more critical in bioanalytical applications for processing and collecting samples. These approaches are predicted to develop more portable and moderately priced biosensors that can meet the needs of modern healthcare services connected to upgrading the quality of life.
